# Bioceramic materials with ion‐mediated multifunctionality for wound healing

**DOI:** 10.1002/SMMD.20220032

**Published:** 2022-12-27

**Authors:** Xiaocheng Wang, Min Tang

**Affiliations:** ^1^ Department of NanoEngineering University of California San Diego San Diego California USA

**Keywords:** bioactive ions, bioceramics, biomaterials, multifunctional wound dressings, wound healing

## Abstract

Regeneration of both anatomic and functional integrity of the skin tissues after injury represents a huge challenge considering the sophisticated healing process and variability of specific wounds. In the past decades, numerous efforts have been made to construct bioceramic‐based wound dressing materials with ion‐mediated multifunctionality for facilitating the healing process. In this review, the state‐of‐the‐art progress on bioceramic materials with ion‐mediated bioactivity for wound healing is summarized. Followed by a brief discussion on the bioceramic materials with ion‐mediated biological activities, the emerging bioceramic‐based materials are highlighted for wound healing applications owing to their ion‐mediated bioactivities, including anti‐infection function, angiogenic activity, improved skin appendage regeneration, antitumor effect, and so on. Finally, concluding remarks and future perspectives of bioceramic‐based wound dressing materials for clinical practice are briefly discussed.

## Graphical abstract

In this review, we provide a brief summary of the state‐of‐the‐art progress on bioceramic materials with ion‐mediated bioactivity for wound healing applications. The emerging bioceramic‐based materials for wound healing are highlighted based on their ion‐mediated bioactivities, including enhanced angiogenesis, antibacterial activity, improved skin appendage regeneration, anti‐tumor effects, wound monitoring, and other multi‐functions.

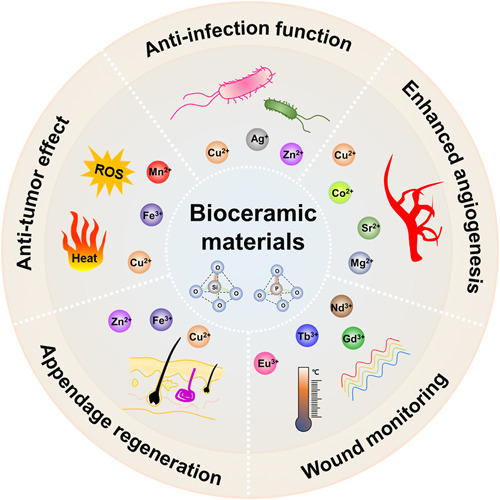

1

**Key points**

- Recent progress on bioceramic materials with ion‐mediated multifunctionality for wound healing.
- Design strategies that are being used to develop bioceramic‐based materials for wound healing.
- Representative ion‐mediated multiple functions, including anti‐infection function, angiogenic activity, improved skin appendage regeneration, antitumor effect, etc.
- Future challenges and perspectives that may lead to the development of bioceramic materials.

## 2 INTRODUCTION

Wound management remains a major healthcare burden worldwide due to the rising prevalence of traumatic injuries and chronic wounds in various pathophysiological conditions.^1^, ^2^, ^3^ Generally, wound healing involves a sequence of highly ordered biological stages, including hemostasis, inflammation, angiogenesis, growth, and re‐epithelialization.^4^, ^5^ However, the wound healing ability is diminished in case of large‐scale trauma or other health complications such as pressure sores, diabetic ulcers, vascular diseases, and aging populations.^6^, ^7^, ^8^ Current medical treatments like gauzes, bandages, cotton wools, and hydrophilic wound dressings commonly aim to provide a physical barrier to shield the wounds from contamination, maintain an appropriate level of humidity, absorb excess exudate at wound sites, and promote the healing process by delivering specific bioactive agents.^9^, ^10^ However, these strategies have limited efficiency in healing large‐scale injuries and chronic wounds, because they primarily depend on the slow and passive wound healing processes. Therefore, a wide range of innovative wound dressing materials with high biological activities and advanced functionalities have been developed to stimulate wound healing and tissue regeneration.^11^, ^12^, ^13^, ^14^, ^15^, ^16^, ^17^ These wound dressings may appear in different physical forms, such as films, sponges, foams, fibrous membranes, patches, hydrogels (sheets, bulks, or injectable ones), gels, creams, ointments, pastes, and so on.^18^ They usually contain antimicrobial drugs, antibiotics, anti‐inflammatory agents, growth factors, cytokines, or other biological components including bioactive ceramic materials.^19^

**FIGURE 1:**
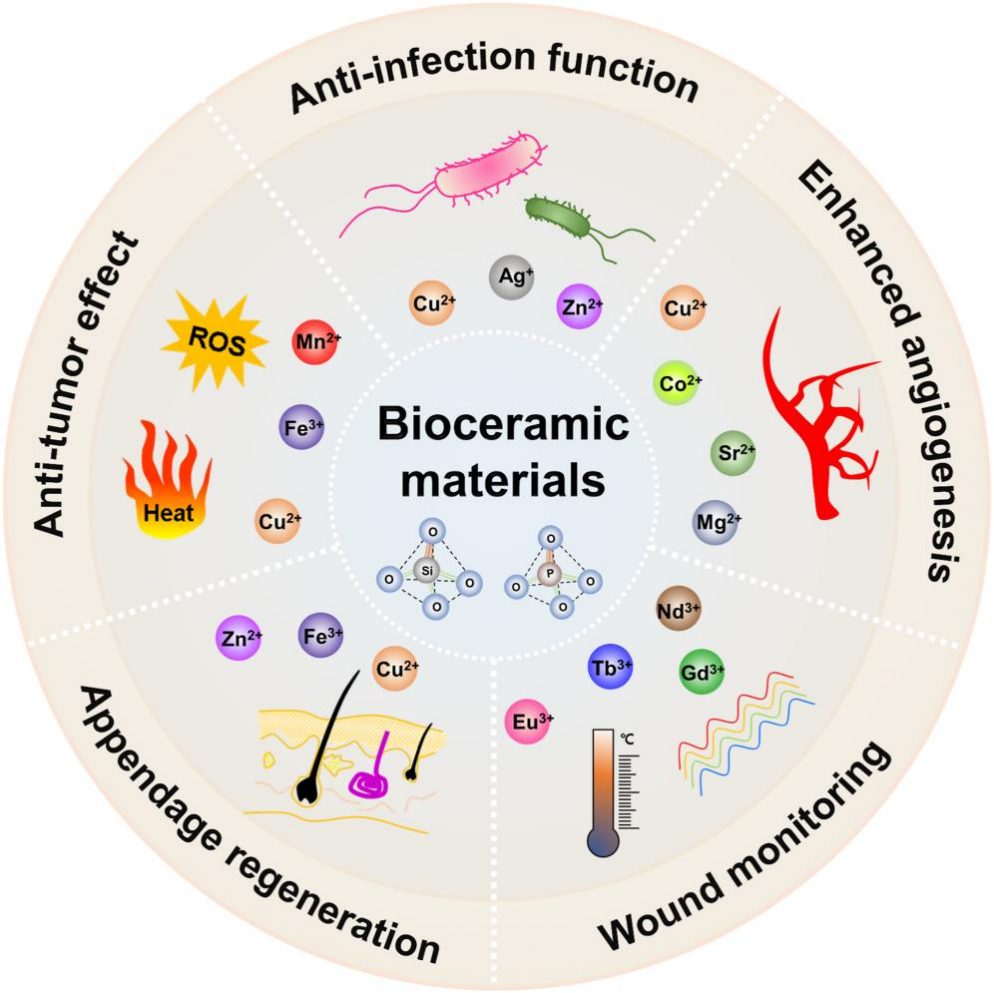
Schematic illustration of bioceramic materials with ion‐mediated multifunctionality for wound healing.

Bioactive ceramics or bioceramics, as a new family of biomaterials, have attracted tremendous attention in regenerative medicine owing to their intrinsic bioactivity and tunable properties.^20^, ^21^, ^22^, ^23^, ^24^ The dissolution ion products from bioceramic‐based materials, for example, calcium (Ca), silicon (Si), magnesium (Mg), copper (Cu) ions, and so on, can provide biochemical cues to control and direct cellular functions through intracellular signaling.^18^, ^24^, ^25^, ^26^ In the past few decades, bioceramic‐based materials with ion‐mediated bioactivity have been widely investigated for facilitating wound healing considering their desirable properties, including antibacterial activity, angiogenic activity, promoting skin appendage regeneration, antitumor effects, immunoregulation, tailing mechanical properties, and so on.^27^, ^28^, ^29^, ^30^, ^31^, ^32^, ^33^, ^34^ In this review, we will focus on the state‐of‐the‐art progress on bioceramic materials with ion‐mediated bioactivity for wound healing applications, as illustrated in Figure 1. Firstly, we will provide an overview of bioceramic materials with ion‐mediated biological activities. Next, we will exemplify the current bioceramic‐based materials for wound healing applications based on their ion‐mediated bioactivities, including anti‐infection function, angiogenic activity, the regenerative capacity of skin appendages, anti‐tumor efficacy, and other multi‐functions. Finally, we will provide concluding remarks and our insights into the bioceramic‐based wound dressing materials for clinical practice.

## 3 BIOCERAMIC MATERIALS WITH ION‐MEDIATED BIOACTIVITY

### 3.1 Overview of bioceramics

The term “bioceramic” is a general term used to cover glasses, glass‐ceramics, and ceramics applied for graft implants and artificial scaffolds.^35^ More specifically, bioceramics typically possess nonmetal structures with high toughness and usually are prepared by sintering of inorganic compounds (e.g., metal oxides) in complex mixtures.^36^ Bioceramics have appeared as one of the most promising biomaterials for tissue engineering. The development of ceramic materials in biological applications has shifted from tissue replacement using bioinert ceramics (e.g., alumina, zirconia, and carbon or nitride‐based ceramics) to tissue repair/regeneration using bioactive ceramics (e.g., bioglasses, bioactive glass‐ceramics, calcium silicate, calcium phosphate, hydroxyapatite ceramics).^37^, ^38^ The main characteristics of bioceramics include thermal and electrical insulation, high brittleness and hardness, corrosion resistance as well as good biocompatibility, biodegradability, and bioactivity.^39^ The bioactivity of bioceramic materials is mainly derived from their tendency to release inorganic ions that are involved in many biological processes.^18^, ^24^, ^26^, ^40^ For instance, the Ca ion plays a significant role in regulating collagen formation and osteogenic differentiation, while Si and P ions are helpful for bone formation in biomineralization.^41^, ^42^, ^43^, ^44^ In addition, the microstructure of bioceramics (e.g., crystallinity, particle size, porosity, and geometry) also plays a critical role in their mechanical and biological properties.^45^, ^46^, ^47^ The architecture design of bioceramic‐based materials at macro, micro, or nano levels is highly essential not only because they provide physical cues for cell‐matrix interactions, but also because they have a great influence on the ionic dissolution rate of bioceramics and their ultimate biofunctions.^48^

### 3.2 Ion‐mediated biological functionality

**FIGURE 2:**
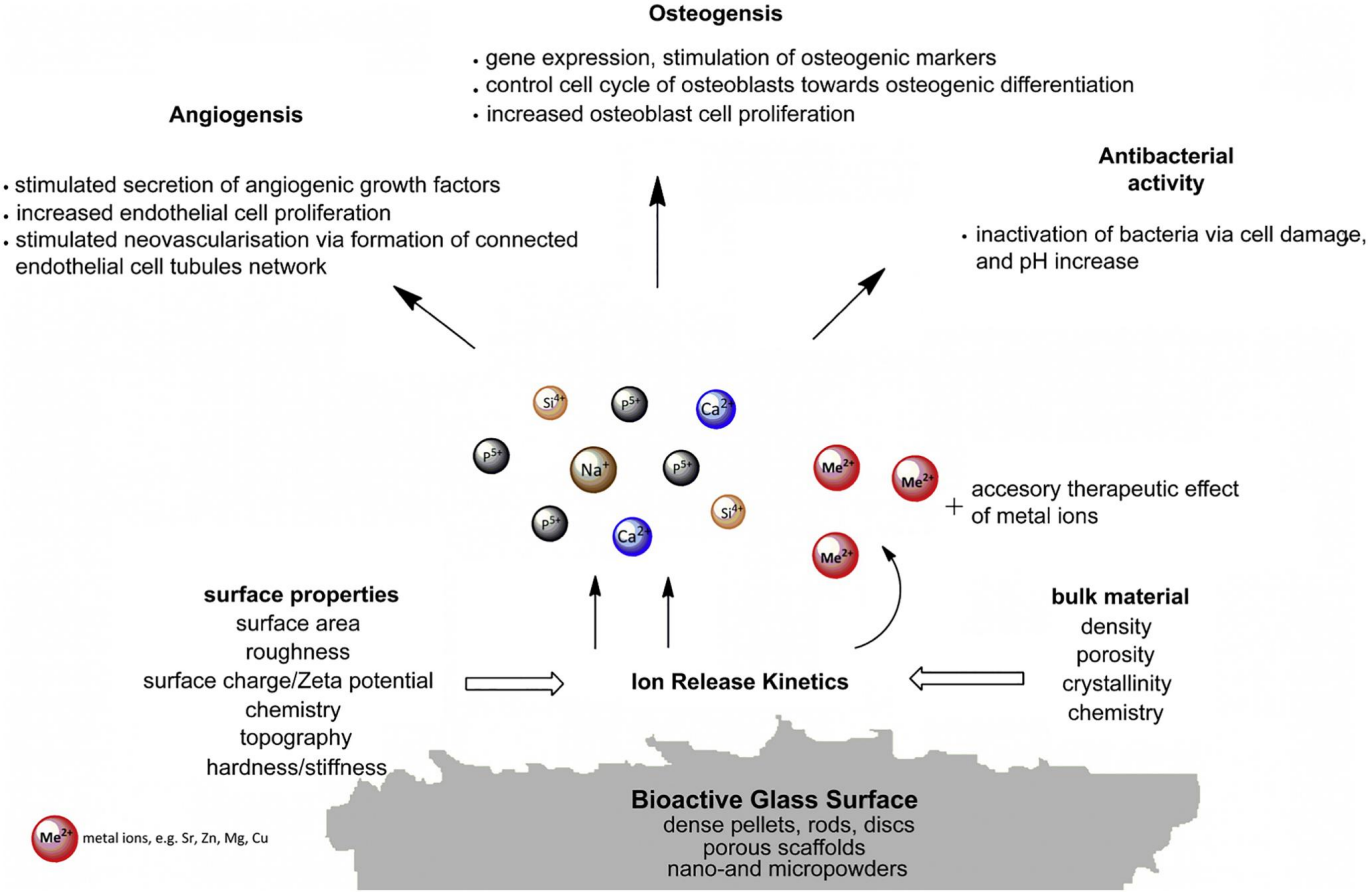
Overview of ion‐mediated biological functions of bioactive glasses. Reproduced with permission.^26^ Copyright 2011, Elsevier.

Bioceramic materials involved in wound healing mainly include noncrystalline bioactive glasses (BGs) and crystalline silicate or phosphate ceramics. Bioceramics are believed to play stimulatory roles in the skin regeneration process owing to their ionic dissolution products. As essential constant elements in the human body, silicon (Si) and phosphorus (P) exhibit positive effects on the formation of blood vessels (i.e., angiogenesis), collagen deposition, and epidermal regeneration during the wound healing process.^49^ Taking BGs as an example, BGs are classified based on their chemical composition into silicate bioactive glasses (SBG), phosphate bioactive glasses (PBG), and borate bioactive glasses (BBG).^18^, ^35^, ^50^ The amorphous glass networks facilitate the sustainable release of bioactive ions with the gradual degradation process of BGs in physiological environments. More importantly, apart from the main chemical components (i.e., Si, Ca, P, B), diverse therapeutic ions (Ag, Au, Li, Sr, Cu, Mg, Zn, Ce, Mo, Mn, Fe, Nd, Eu, etc.) can be integrated into their flexible glass network to improve the biological properties of BG such as angiogenesis, osteogenesis, and antibacterial effects.^25^, ^27^, ^30^, ^33^, ^51^, ^52^, ^53^, ^54^, ^55^, ^56^, ^57^, ^58^, ^59^, ^60^, ^61^, ^62^, ^63^, ^64^, ^65^ The biological responses to ionic dissolution products of BGs are schemed in Figure 2.^26^

Generally, the metallic ions are important components of many enzymes and function as cofactors that stimulate metabolic reactions associated with cell signaling pathways during tissue regeneration.^26^, ^66^, ^67^, ^68^ For example, Ca ion is a key factor in the hemostatic phase, and also acts as an intracellular modulator and extracellular regulator of epidermal migration, proliferation, differentiation, and collagen synthesis in the wound healing process.^69^, ^70^, ^71^, ^72^, ^73^ Mg ion plays a regulatory role in epidermal proliferation, differentiation, and skin barrier functions.^74^, ^75^ Sr ion has been reported to promote angiogenesis and suppress inflammation in skin wound treatments.^76^, ^77^, ^78^ Cu ion is well‐known for its significant role in angiogenesis by regulating the expression of proangiogenic factors such as vascular endothelial growth factor (VEGF) and transforming growth factor‐β (TGF‐β) as well as the hypoxia‐inducible factor (HIF‐1α) to stimulate skin wound healing.^25^, ^27^, ^79^, ^80^ In addition, Cu ion has antibacterial and anti‐inflammatory properties. Zn ion mainly functions as regulators of many skin cells, including keratinocytes, melanocytes, Langerhans cells, dendritic cells, and so on, exhibiting antioxidant, antimicrobial, and angiogenic properties in the treatment of wounds.^53^, ^81^, ^82^, ^83^ These ion‐mediated biological effects of bioceramics make them an ideal choice for wound healing applications. It is worth noting that the biological effects of bioceramic ions function in a dose‐dependent manner, and they may be toxic if used in a fairly high dose.^84^, ^85^

### 3.3 Design of bioceramic‐based wound dressing materials

**FIGURE 3:**
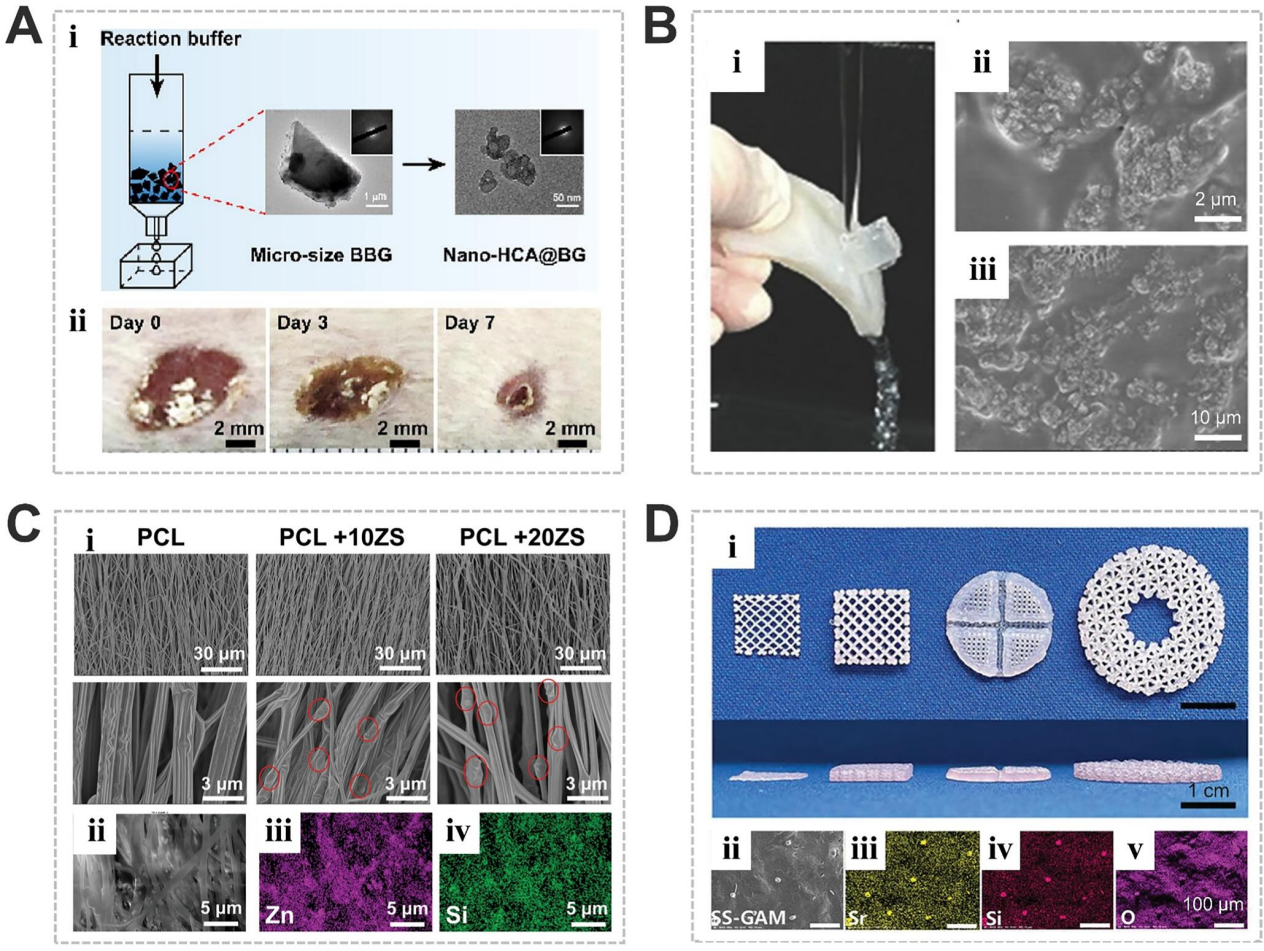
Representative bioceramic‐based materials used for skin wound treatment. (A) Bioceramic particles or powders: (i) Preparation of nanosized CO_3_^2−^‐containing hydroxyapatite (HCA)‐coated BBG (nano‐HCA@BG) and (ii) evaluation of the wound healing effect of nano‐HCA@BG powders in mouse skin defects. Reproduced with permission.^86^ Copyright 2021, Elsevier. (B) Bioceramics‐incorporated composite hydrogels: (i) Photographs showing the strong adhesion of the hydroxyapatite (HAp)‐incorporated poly(N‐acryloyl 2‐glycine) (PACG‐HAp) hydrogel to porcine skin under water flushing; and (ii, iii) SEM images showing the HAp nanoparticles uniformly dispersed in the PACG‐HAp hydrogel networks. Reproduced with permission.^72^ Copyright 2018, John Wiley and Sons. (C) Bioceramics‐incorporated electrospun membranes: (i) SEM images of zinc silicate (ZS) nanoparticles‐incorporated poly(ε‐caprolactone) (PCL) electrospun nanofibers; (ii) SEM image and corresponding EDS element mappings of (iii) element Zn and (iv) element Si of PCL+20ZS scaffolds. Reproduced with permission.^53^ Copyright 2022, John Wiley and Sons. (D) 3D printed bioceramics‐containing scaffolds: (i) 3D printed strontium silicate‐containing Gellan Gum hydrogel (SS‐GG) scaffolds with various design patterns; (ii) SEM image of the surface of freeze‐dried SS‐GAM scaffold and corresponding EDS elemental mapping of (iii) element Sr, (iv) element Si, and (v) element O. Reproduced with permission.^54^ Copyright 2021, John Wiley and Sons.

Benefiting from their high bioactivities and multifunctionalities, the bioceramic materials can be either applied alone in the forms of fine powders or nanoparticle suspension,^27^, ^80^, ^86^, ^87^ or employed as the functional counterparts in composite wound dressing materials in the forms of bioceramic‐incorporated hydrogels,^29^, ^30^, ^32^, ^59^, ^61^, ^72^, ^88^, ^89^ films or membranes,^28^, ^31^, ^33^, ^53^, ^90^, ^91^, ^92^, ^93^, ^94^, ^95^ sponges,^73^, ^96^, ^97^ porous 3D scaffolds,^25^, ^52^, ^54^, ^98^ and so on (Figure 3). For example, Zhao et al. prepared a kind of Cu‐BBG microfibers with various Cu‐doping contents and evaluated their wound healing ability both in vitro and in vivo.^27^ The ionic dissolution products (e.g., Ca, B, and Cu ions) not only promoted migration, tube formation, and VEGF expression of human umbilical vein endothelial cells, but also stimulated the expression of several angiogenesis‐related genes (bFGF, VEGF, and PDGF) of fibroblasts. Compared to undoped fibers, Cu‐doped fibers showed an improved ability to promote angiogenesis for healing full‐thickness skin defects in rats. For another example, Chen et al. coated the nanosized BBG with CO_3_^2−^‐containing amorphous hydroxyapatite (HCA) to improve the biodegradability and biocompatibility of BBG (Figure 3A).^86^ Compared with the uncoated BBG powders, the nano‐HCA@BG particles showed a controllable ion‐release ability, contributing to the promoted cell proliferation in vitro and better skin wound healing effects in vivo.

When bioceramic particles/powders are directly applied to wound beds, their inherent brittleness and hardness are likely to induce acute inflammatory reactions and cell damage.^55^, ^88^, ^99^ In this regard, bioceramic‐based wound dressing materials are more effective and flexible strategies for wound healing applications. For example, Cui et al. designed an organic‐inorganic hybrid hydrogel with high‐strength self‐adhesive properties by incorporating hydroxyapatite (HAp) into the poly(N‐acryloyl 2‐glycine) (PACG) hydrogel networks (Figure 3B). The HAp not only strengthened the supramolecular polymer hydrogels by the ionic crosslinking of Ca^2+^ ions to the carboxyl groups on the PACG chains, but also improved the biocompatibility and promoted wound healing with instant hemostatic effects.^72^ In tissue regeneration fields, it has been accepted that the porous scaffolds with nano/microscale biomimetic architectures could bring about a better tissue regeneration capacity for offering structural cues for new tissue ingrowth and construction.^53^, ^54^, ^100^ For example, Zhang et al. developed zinc‐silicate‐incorporated nanofibrous scaffolds via an electrospinning process (Figure 3C).^53^ The bioceramic‐nanoparticle‐embedded nanofibrous scaffolds could maintain a sustained release of Zn and Si bioactive ions, which accelerated the formation of blood vessels and nerve fibers in second‐degree skin burn defects. In another example, Ma et al. utilized strontium silicate (SS) particles as cell‐induced factors for angiogenesis and integrated the particles into the cell containing bioprinting inks.^54^ Such bioceramic‐containing multicellular biohybrid scaffolds exhibited excellent angiogenic activity and significantly accelerated skin wound healing in vivo (Figure 3D).

Given the significant characteristics mentioned above, such as good biocompatibility, biodegradability, flexible chemical formulation, ease of preparation, and tunable ion‐mediated multifunctionality to accelerate wound healing, the bioceramics have attracted many researchers to employ them as multifunctional wound dressing materials for diverse wound healing applications.

## 4 MULTI‐FUNCTIONAL BIOCERAMIC MATERIALS FOR WOUND HEALING APPLICATIONS

### 4.1 Anti‐infection function

Skin acts as the first line of defense that protects internal tissues from the external damage and invasion of pathogenic organisms.^101^ Once the skin is damaged, bacteria may invade into injured tissues, leading to impaired wound healing marked by excessive inflammation and persistent infections.^102^, ^103^, ^104^ Traditional antibiotics are commonly used in clinics to either kill or inhibit the growth of pathogenic microorganisms, while their antimicrobial efficacy is compromised concerning the increasing levels of antibiotic resistance and side effects of overused antibiotics.^105^, ^106^, ^107^, ^108^ Therefore, it is highly expected to develop new wound dressing materials with enhanced antimicrobial activity for wound healing.

Apart from the excellent tissue regenerative capacity, some bioceramic materials such as silicate or bioactive glasses possess intrinsic antibacterial activity owing to the alkaline microenvironment caused by the release of alkaline metal ions during their degradation process.^109^, ^110^ However, such pH‐dependent antibacterial activity is limited for an effective anti‐infection functionality. For bioceramic materials, enhanced wound healing ability and anti‐infection function can be achieved by tailoring the chemical compositions to incorporating specific inorganic elements with intrinsic antibacterial effects such as Ag, Au, Cu, Zn, Mo, Ga, Ce ions.^25^, ^111^, ^112^, ^113^, ^114^, ^115^, ^116^ For example, Li et al. prepared an bioactive hardystonite (Ca_2_ZnSi_2_O_7_) ceramic‐incorporated hydrogel via double ion cross‐linking of Ca^2+^ and Zn^2+^ to alginate polymer networks.^117^ Their results showed that the released Si ions from the composite hydrogel could promote new blood vessel formation and accelerate wound healing, while the released Zn ions inhibited the growth of bacteria.

**FIGURE 4:**
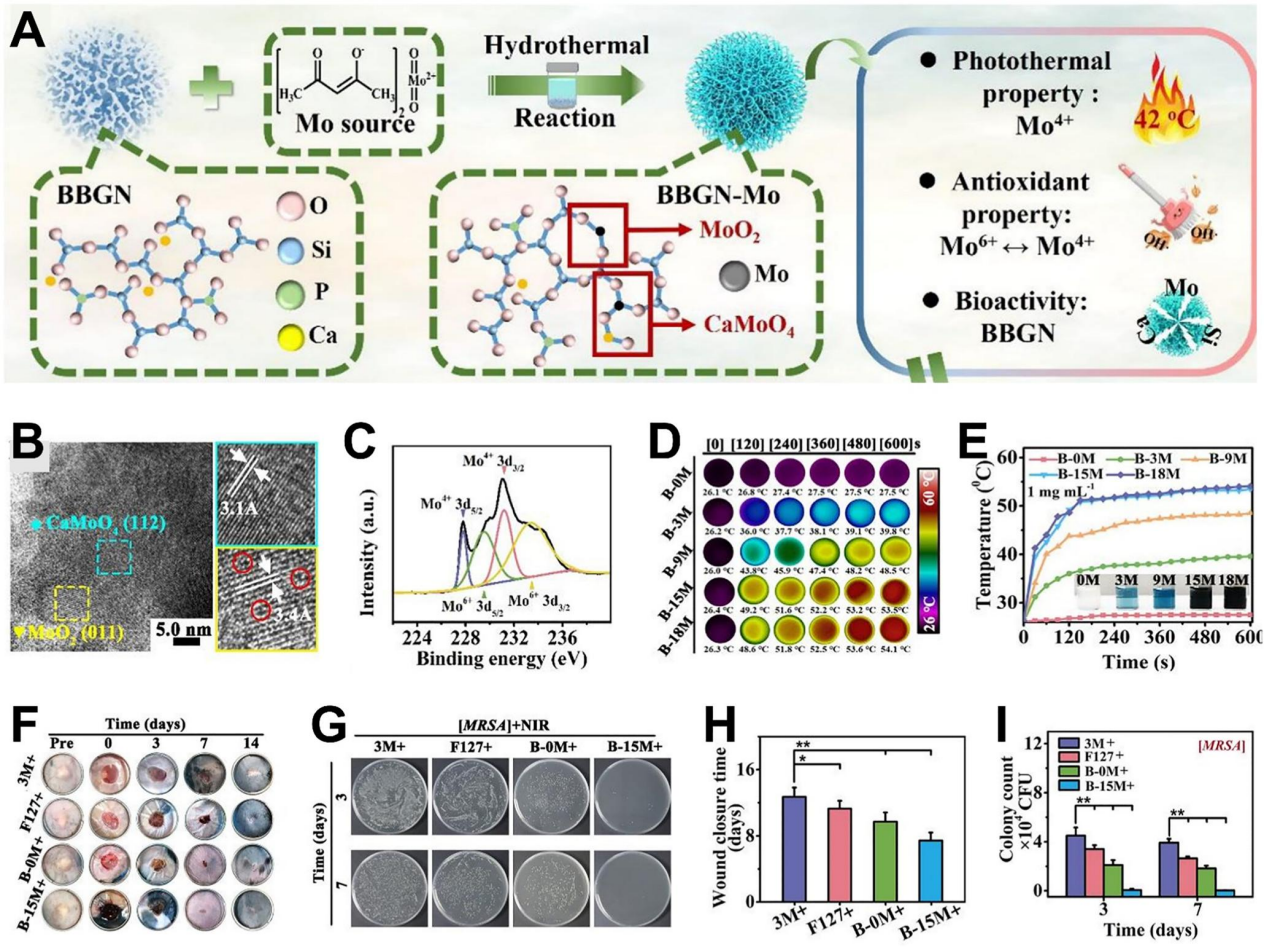
Bioceramic‐based wound dressings for effective infection therapy and skin wound healing. (A) Synthesis and structure illustration of BBGN‐Mo (B‐M) with multifunctional activities. (B) High‐resolution TEM of B‐M (blue pane: CaMoO_4_ nanocrystal, yellow pane: MoO_2_ nanocrystal). (C) XPS spectra of Mo 3d of B‐M. (D) Real‐time infrared thermal images of B‐M samples. (E) Photothermal heating curves (808 nm) and photographs (inset) of various B‐M samples at 1 mg/ml. (F) Photographs of MRSA‐infected skin wounds during 14 d of treatment with different samples under an NIR irradiation for 10 min. (G) Changes in wound size after various treatments (*n* = 6). (H, I) Wound tissue‐derived bacteria colonies (H) and corresponding statistical data of colonies (I) against MRSA after incubation for 3 days (*n* = 6). *p < 0.05; **p < 0.01. Reproduced with permission.^30^ Copyright 2021, American Chemical Society.

In addition to the ion‐induced antibacterial effect, photothermal therapy (PTT) has recently been utilized to kill bacteria via physical heating.^30^, ^118^, ^119^ In one case, a multifunctional composite hydrogel combined Cu‐doped bioceramics with polydopamine (PDA) was designed for infectious skin wound healing.^120^ In this system, the photothermal heating effect of both PDA and Cu ions showed high efficiency and long‐term inhibition of methicillin‐resistant *Escherichia coli* and *Staphylococcus aureus*. Additionally, the Cu ion acted as a bioactive agent to stimulate angiogenesis for wound healing. Similar to Cu ions, Mo ions also exhibited an excellent PPT effect upon near‐infrared (NIR) laser irradiation, which could be used for photothermal anti‐infection therapy. For example, Lei group report a multifunctional bioactive Si‐Ca‐P‐Mo glass‐ceramic nanoparticle (BBGN‐Mo or B‐M) for effective infection therapy and skin wound healing (Figure 4).^30^ The Mo‐containing bioceramic nanoparticles were prepared via a hydrothermal reaction between branched spherical BBGN and C_10_H_14_MoO_6_ (Figure 4A). Compared with BBGM (B‐0M), the BBGN‐Mo displayed the following major functionalities: (i) excellent photothermal and antioxidant activity due to the existence of oxygen vacancies and free electrons (Figure 4B,C); (ii) photothermal performance with “biocompatible photothermal temperature” achieved by controlling the ratio of Mo^4+^ to Mo^6+^ (Figure 4D,E); (iii) effective antibacterial efficiency for infected wounds after surgery (Figure 4F–I); (iv) selective photothermal anticancer activity to inhibit the tumor reoccurrence without obvious side effect on healthy tissues for healing the tumor resection defects; and (v) a significantly higher wound healing rate in vivo with more skin appendage formation and enhanced re‐epithelialization via releasing bioactive ions.

### 4.2 Enhanced angiogenesis

Angiogenesis plays an important role in the wound healing process due to the function of delivering nutrients and oxygen for new tissue formation.^121^, ^122^, ^123^ Enhanced in vitro and in vivo angiogenic capacity has been extensively demonstrated by the ionic dissolution products from bioceramic materials.^29^, ^49^, ^100^, ^124^, ^125^ For example, Chang group previously reported nanosized‐SBG‐coated hierarchically micro‐patterned nanofibrous scaffolds for accelerating wound healing.^100^ The hierarchical micro/nanostructures of nanofibrous scaffolds together with the release of Ca, Mg, and Si ions from the SBG coating synergistically promoted angiogenesis, and improved in vivo wound closure rate and healing quality. Apart from amorphous glasses, bioceramic crystals such as Nagelschmidtite nanoparticles (Ca_7_P_2_Si_2_O_16_) have been demonstrated effective for healing diabetic wounds by promoting vascularization, collagen synthesis, and epidermal regeneration.^49^

**FIGURE 5:**
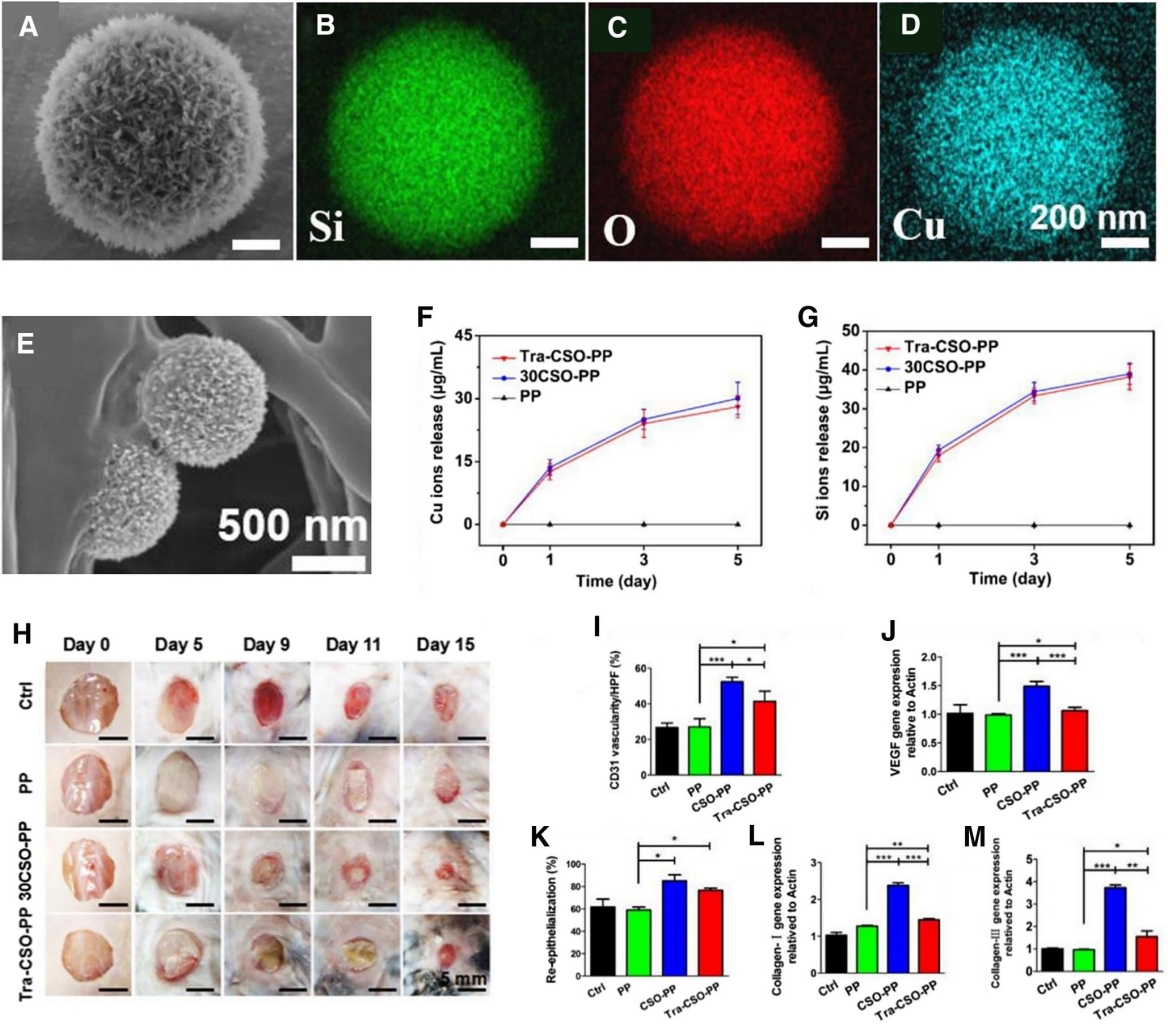
Bioceramic‐based wound dressings for enhanced angiogenesis. (A) SEM image and (B–D) corresponding EDX element mappings (Si, O, and Cu, respectively) of copper silicate hollow microspheres (CSO HMSs). (E) SEM image of CSO HMSs‐incorporated electrospun (CSO‐PP) scaffold. The release profiles of (F) Cu and (G) Si ions in Dulbecco's modified Eagle's medium after soaking CSO‐PP scaffolds for 5 days. (H) Representative skin wound photographs on days 0, 5, 9, 11, and 15. Quantitative analysis of (I) CD31‐positive blood vessels, (J) gene expression of VEGF, (K) re‐epithelialization, (L) expression of collagen III, and (M) collagen I on day 15 (*p < 0.05, **p < 0.01, ***p < 0.001). The CSO‐PP scaffolds upregulated expression of CD31, VEGF, and collagen I/III, indicating expedited revascularization and re‐epithelialization by the stimulatory effect of CSO HMSs. Reproduced with permission.^28^ Copyright 2018, American Chemical Society.

Besides antibacterial effect, Cu ion is also known for its stimulatory role in angiogenesis.^25^, ^27^, ^79^ Cu‐containing bioceramic materials have been incorporated into composite wound dressings and demonstrated effective for enhanced angiogenesis in wound healing applications.^28^, ^55^, ^80^ For example, Wu group previously used Cu‐containing BGs to coat the eggshell membranes for both antibacterial performance and angiogenic capacity.^55^ Their results showed that the bioceramic‐modified eggshell membranes could not only improve angiogenesis‐related gene expression in vitro, but also accelerate the revascularization in vivo. More recently, we prepared a bioceramic‐particle‐incorporated electrospun membrane by using copper silicate hollow microspheres (CSO HMSs) as the source of Cu and Si ions for healing skin tumor‐induced wounds (Figure 5).^28^ CSO HMSs were synthesized through a hydrothermal process and displayed a hollow microstructure with uniformly distributed O, Si, and Cu elements in the whole microspheres (Figure 5A–D). For wound healing application, we incorporated the CSO HMSs into electrospun fibrous scaffolds (Figure 5E) and further confirmed that Si and Cu ions could be gradually released from the CSO‐incorporated electrospun scaffolds (Figure 5F,G). The ion‐mediated tissue regenerative bioactivity of such bioceramic scaffolds was demonstrated to accelerate diabetic wound healing in mice by promoting angiogenesis and re‐epithelialization (Figure 5H–M).

### 4.3 Stimulation of skin appendage regeneration

Intact skin is composed of the epidermis, dermis, and skin appendages, including hair follicles, sweat glands, and sebaceous glands.^126^ As skin appendages are essential for the biological and physiological functions of skin tissues, complete wound healing should include skin function recovery and structural integrity together with the regeneration of skin appendages.^127^ However, for adult mammals, wound healing typically results in scar tissues without skin appendages. Therefore, many efforts have been made in the development of tissue‐engineered biomaterials for scarless healing with appendage regeneration.^22^, ^128^, ^129^, ^130^

**FIGURE 6:**
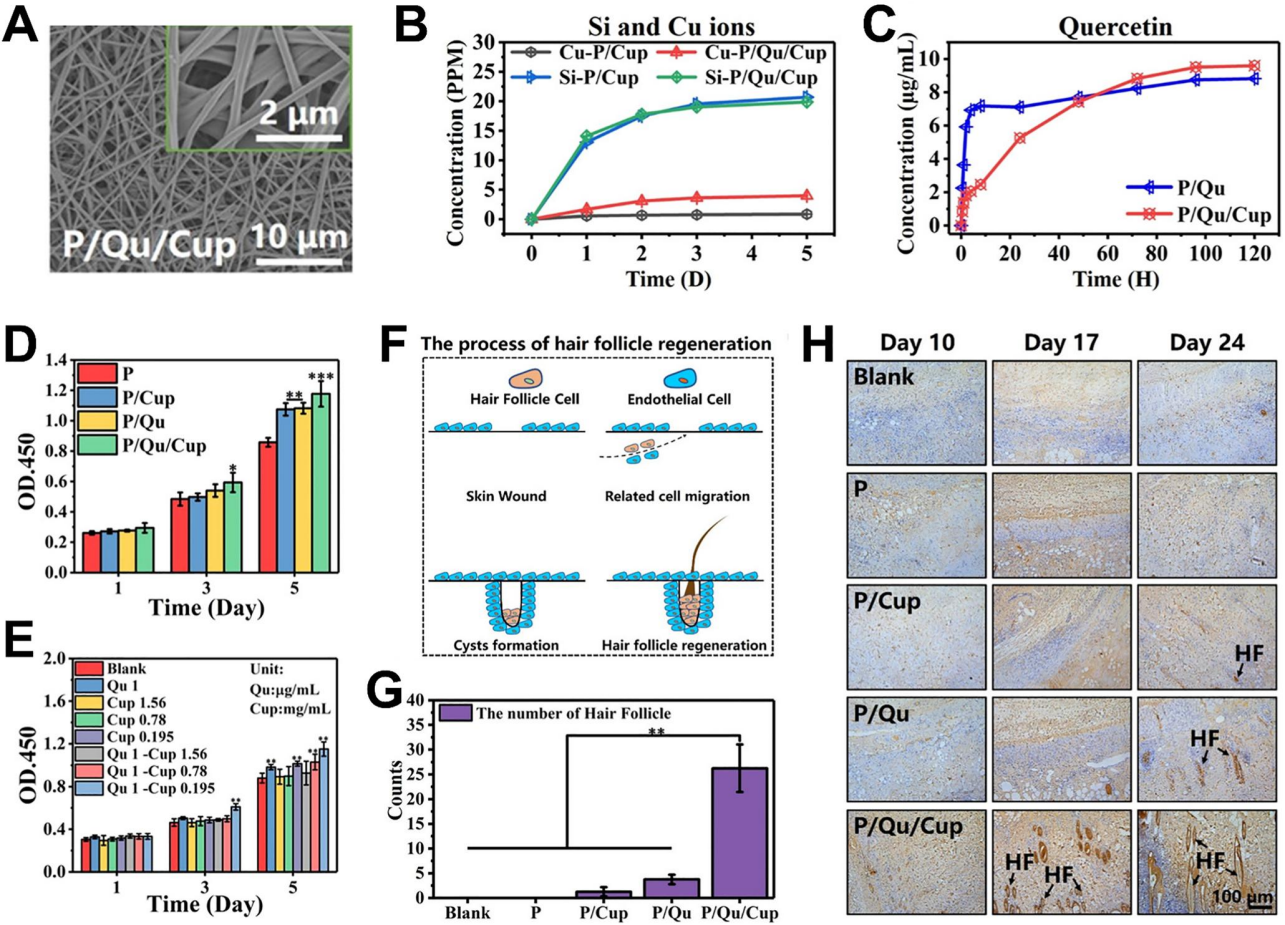
Bioceramic‐based wound dressings for hair regeneration in burned skin. (A) SEM images of the quercetin (Qu) and bioceramic (CaCuSi_4_O_10_) incorporated (P/Qu/Cup) electrospun composite fibrous membranes. (B, C) Release of Si and Cu ions and Qu in P/Cup, P/Qu, and P/Qu/Cup composite membranes. (D, E) Proliferation of human hair follicle dermal papilla cells (HHDPCs) cultured with (D) Qu‐Cup composite fiber membranes and (E) Qu/Cup extracts. (F) Schematic illustration of hair follicle regeneration process. (G) Number of hair follicles on Day 24. **p < 0.01, *n* = 6. (H) Images of immunohistochemical staining for cytokeratin 19 on Day 10, 17 and 24. HF indicated the newly‐formed hair follicles. Reproduced with permission.^94^ Copyright 2020, American Chemical Society.

Bioceramic‐based materials with ion‐mediated bioactive properties have shown great potential in promoting optimal wound healing with the regeneration of skin appendages such as hair follicles. Recently, Chang group fabricated a series of bioceramic‐incorporated electrospun fibrous scaffolds for stimulation of skin follicle regeneration.^33^, ^56^, ^57^, ^94^ In one case, they integrated the cuprorivaite (CaCuSi_4_O_10_) particles and quercetin (Qu) into electrospun fibers (Figure 6A).^94^ Bioactive Cu and Si ions as well as Qu‐Cu chelates could be released from the composite membranes (Figure 6B,C), contributing to enhanced proliferative capacity of hair follicle dermal papilla cells (Figure 6D,E) and synergistic stimulatory effects on the hair follicle regeneration and skin tissue reconstruction (Figure 6F–H). Similarly, they also demonstrated the synergistic effect of Zn‐Si^33^, ^57^ or Fe‐Si^56^ bioceramic incorporated biopolymer fibers to promote hair follicle regeneration during wound healing.

### 4.4 Anti‐tumor effect and simultaneous wound healing

Skin cancer is one of the most common frequent solid cancers with an estimated annual increase of 3%–7% in White populations.^131^, ^132^ The most common treatment in clinics is surgical resection, followed by wound coverage with gauze, bandages, and/or cotton wools.^133^, ^134^, ^135^ In this case, the large‐sized skin wounds are difficult to heal, especially for patients with diabetes or other vascular diseases. Besides, the remaining tumor cells are likely to cause cancer recurrence.^92^, ^98^ Therefore, it is desirable to construct bifunctional wound dressing materials to eliminate skin tumors and simultaneously to heal the tumor‐induced wounds.

**FIGURE 7:**
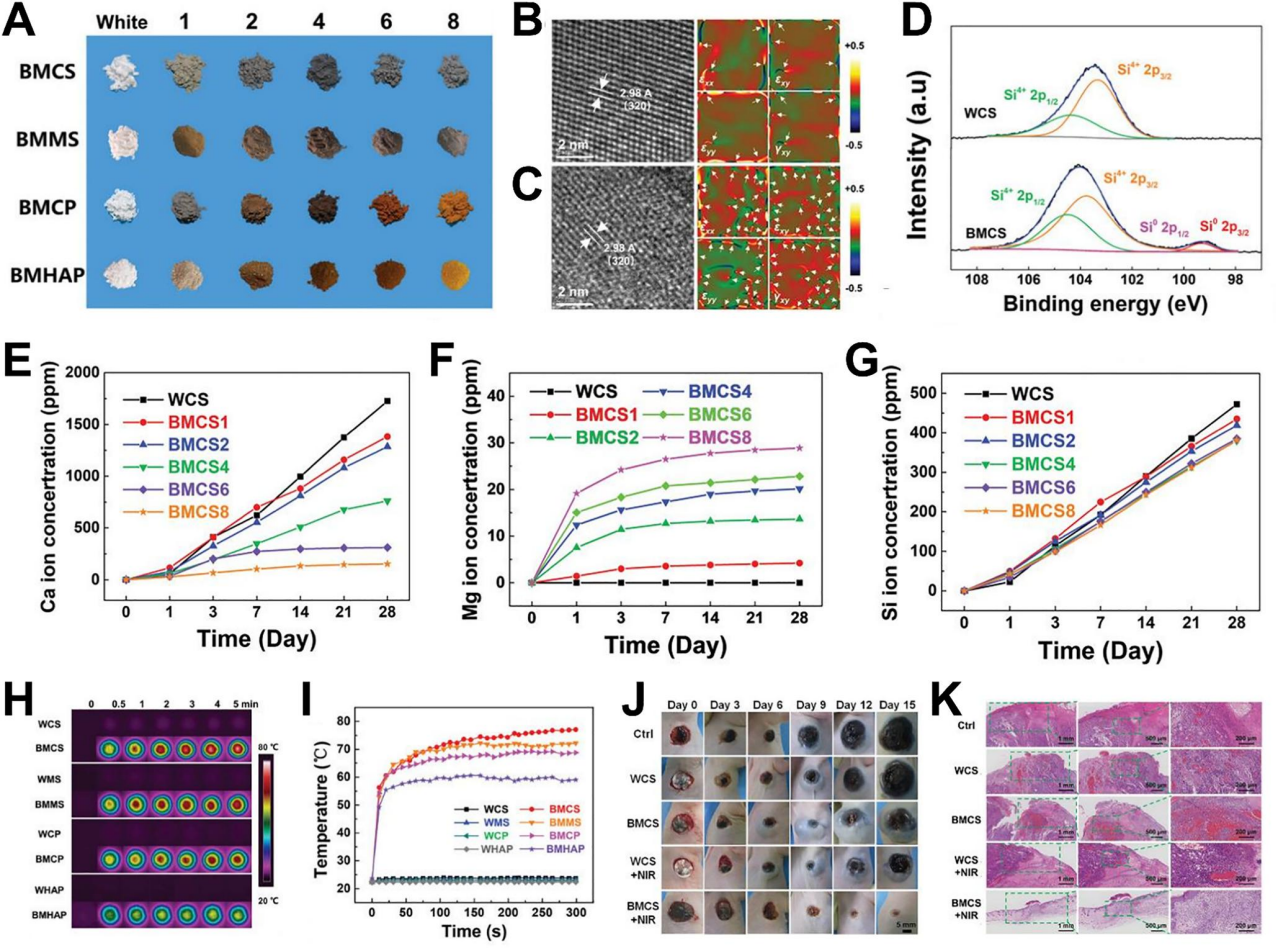
Black bioceramic‐based wound dressings for photothermal tumor therapy and wound healing. (A) Photographs of black CaSiO_3_ (BMCS), MgSiO_3_ (BMMS), Ca_3_(PO_4_)_2_ (BMCP), and Ca_5_(PO_4_)_3_(OH) (BMHAP) powders prepared by magnesium thermal reaction with increasing contents of Mg powders as compared to the pristine white bioceramics. (B, C) High‐resolution TEM image and corresponding geometric phase analysis (GPA) of white CaSiO_3_ (WCS) (B) and BMCS (C) bioceramic powders. The white arrows indicate the presence of misfit dislocations. (D) High‐resolution XPS spectra of Si 2p for WCS and BMCS powders. (E–G) Cumulative amounts of Ca (E), Mg (F), and Si (G) release at each time point from various black BMCS bioceramic powders treated with different amounts of Mg (i.e., BMCS1, BMCS2, BMCS4, BMCS6, and BMCS8) into Tris‐HCl solution at 37°C. (H) Real‐time infrared thermal images and (I) corresponding heating curves of BMCS, BMMS, BMCP, and BMHAP bioceramics as compared to the pristine white bioceramics (WCS, WMS, WCP, WHAP) under NIR irradiation. (J) Representative photographs of skin wounds in skin‐tumor‐bearing mice on days 0, 3, 6, 9, 12, and 15. (K) H&E staining of sectioned skin‐tumor‐induced skin wounds from different groups on day 15. Reproduced with permission.^93^ Copyright 2020, John Wiley and Sons.

Over the past decades, bifunctional wound dressings based on a variety of metal elements (e.g., Cu, Fe, Mn, Eu, Gd)‐meditated bioceramic materials have been designed for tissue regeneration and simultaneous tumor therapy like phototherapy (PTT or PDT).^31^, ^32^, ^61^, ^87^, ^89^, ^91^, ^93^, ^136^ For example, Cu‐containing bioceramics have been employed as photothermal agents owing to their efficient heat generation ability, which is derived from the d‐d electron transition of Cu ions induced by NIR irradiation.^28^, ^31^, ^136^ In addition to the PPT effects, Fe‐containing bioceramics could release Fe ions to catalyze the occurrence of Fenton reaction, thereby generating •OH to achieve tumor microenvironment targeted chemodynamic therapy.^89^ In our recent work, we proposed innovative bioceramic materials denoted as “black bioceramics,” which extended the applications of traditional bioceramic materials from tissue regeneration to tumor therapy (Figure 7).^93^ The black bioceramics were synthesized from traditional white ceramics after magnesium thermal reduction. In our experiment, traditional phosphate‐based (Ca_3_(PO_4_)_2_, Ca_5_(PO_4_)_3_(OH)) and silicate‐based (CaSiO_3_, MgSiO_3_) powders were transformed into black bioceramics, as shown in Figure 7A. Owing to the introduction of structural defects and oxygen vacancies after magnesium thermal reduction (Figure 7B–D), the black bioceramics could effectively release Ca and Si ions along with extra Mg ions in a controllable manner (Figure 7E–G). Notably, with the increasing Mg used in the reduction reaction, the obtained black bioceramic powders could gradually release more Mg ions but fewer Ca ions in specific periods. In addition, black bioceramics could be easily heated up under NIR irradiation at a low laser intensity (Figure 7H,I), resulting in excellent PTT therapeutic effects in skin‐tumor‐bearing mice (Figure 7J,K). Meanwhile, black bioceramic‐based materials could significantly improve the tissue regenerative ability for skin wound healing in mice. These fascinating results indicated the feasibility of using single black bioceramic materials for tumor therapy and tissue regeneration, which not only extends the biomedical potential of bioceramics, but also represents a promising direction for multi‐functional biomaterials.

### 4.5 Other multi‐functions

Wound fluid management is another challenge in wound healing, especially for diabetic ulcer wounds with excessive exudates.^137^, ^138^, ^139^ Recently, engineered Janus amphipathic wound dressings have been developed with anti‐adhesion abilities and unidirectional water transfer function to accelerate wound healing.^95^, ^138^, ^140^, ^141^, ^142^ Besides wound drainage, it is also necessary for stimulating angiogenesis in chronic wound healing. Therefore, Bao et al. designed a multifunctional composite wound dressing membrane, consisting of a modified Janus membrane with a BG‐containing bioactive layer for promoting angiogenesis and a superabsorbent layer for effective water absorption during wound healing.^95^ Compared with traditional Janus membranes, this modified Janus membrane showed unique bi‐directional fluid transport properties, which allowed large amounts of exudate to be pumped from wounds rapidly while a small number of bioactive ions transported back to wound beds.

**FIGURE 8:**
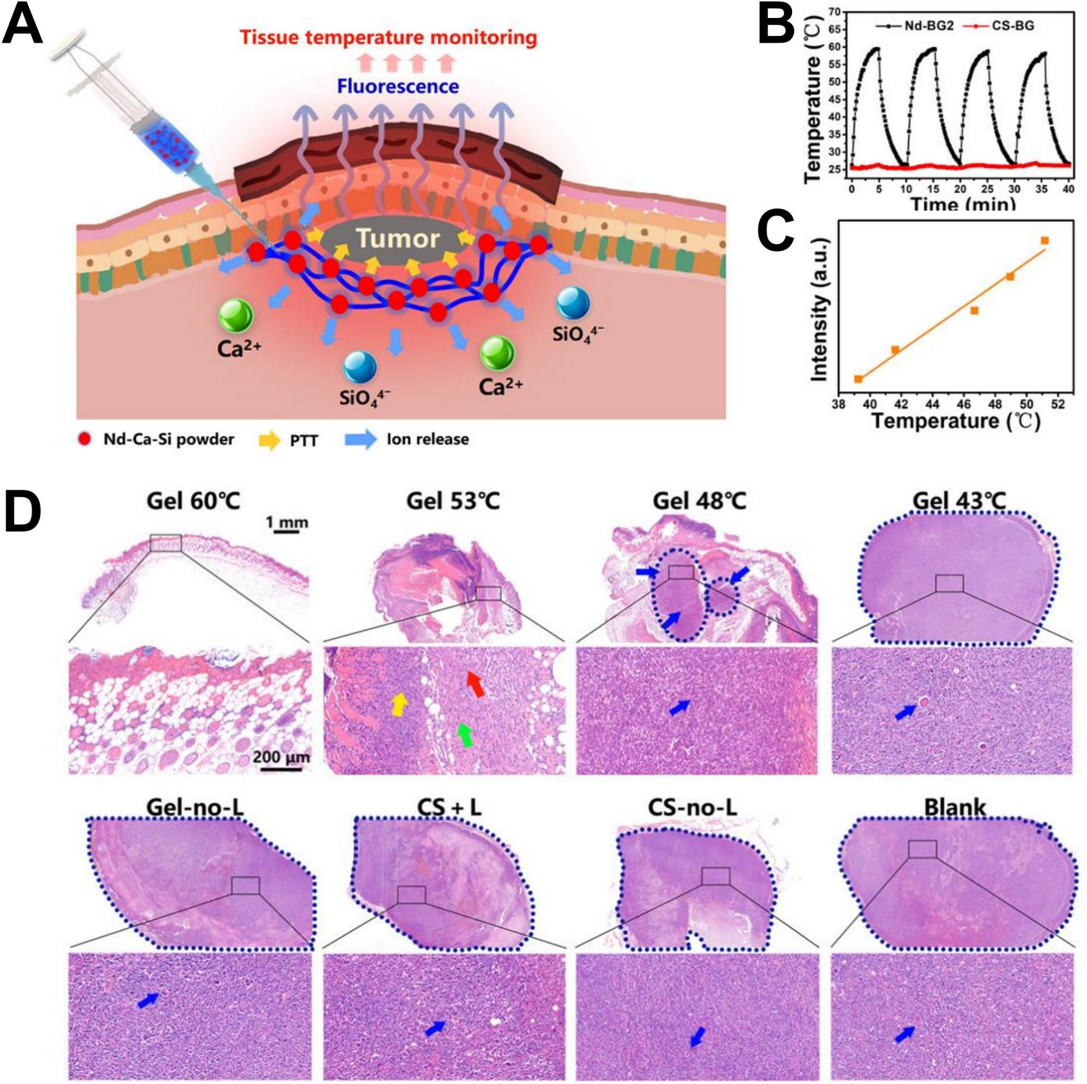
Multifunctional bioceramic‐based wound dressings for in situ temperature monitoring, photothermal therapy, and wound healing. (A) Schematic showing the injectable Nd‐Ca‐Si bioglass/alginate composite hydrogel for in vivo tumor elimination, determining optimal PTT temperature by fluorescence thermometry and repairing heat‐damaged normal tissues by releasing bioactive Ca and Si ions. (B) Heating curves of the Nd‐BG2 and CS‐BG composite hydrogels under 808‐nm laser irradiation. (C) Linear fitting relationship between 1062‐nm fluorescence intensity and temperature for Nd‐BG2 composite hydrogels. (D) H&E staining images of skin tumor tissues after different temperature treatments (blue arrow, tumor tissues; red arrow, fibroblasts; yellow arrow, inflammatory cells; green arrow, new blood capillary). Reproduced with permission.^59^ Copyright 2020, The Authors, published by AAAS.

More recently, smart wound dressings have been developed to monitor the change of physical and chemical parameters at wound sites, including humidity, temperature, pH, reactive oxygen species, enzymes, growth factors, etc.^14^, ^143^ In one case, Chang group reported a multifunctional bioceramic‐based composite hydrogel dressing for determining the optimal temperature in vivo during PTT tumor elimination via Nd‐ion‐mediated fluorescence thermometry, and simultaneously healing the heat‐damaged normal tissues by releasing bioactive Ca and Si ions (Figure 8A).^59^ They first prepared Nd‐doping calcium‐silicon‐based bioactive ceramic (Nd‐BG) materials, which had unique photothermal effects and fluorescence characteristics different from non‐Nd‐doping CS‐BG (Figure 8B,C). Subsequently, they incorporated the Nd‐BG glass into an alginate composite hydrogel and determined the optimal PTT temperature at 53°C in mouse tumor models (Figure 8D). Under this safe temperature, the tumor was completely eliminated without causing damage to surrounding normal tissues. In addition, the composite hydrogel could release bioactive ions to repair the PTT‐damaged tissues. This study suggested that bioceramic materials with photothermal function, wound healing bioactivity, and unique temperature monitoring may satisfy the multi‐functional needs of practical wound treatments.

## 5 SUMMARY AND OUTLOOKS

In this review, we provide a brief summary of the state‐of‐the‐art progress on bioceramic materials with ion‐mediated bioactivity for wound healing applications. We first make a critical discussion on bioceramic materials with ion‐mediated biological activities. Next, we highlighted the emerging bioceramic‐based materials for wound healing applications owing to their ion‐mediated bioactivities, including enhanced angiogenesis, antibacterial activity, improved skin appendage regeneration, anti‐tumor effects, and other multi‐functions. Such multi‐functional bioceramic‐based materials have emerged as one of the most promising wound dressing materials in clinical aspects, and the ion‐mediated wound healing strategy is anticipated to extend the use of bioceramic materials in skin tissue regeneration from the laboratory into the clinic. Since the first bioactive glass invented by Larry Hench in 1969, commercial products derived from bioactive ceramics have been used clinically to treat bone‐ or tooth‐related issues.^35^, ^63^ For example, the bioactive glass S53P4 has been successfully used in clinic as an antibacterial bone substitute with bone‐bonding and osteostimulative properties,^144^ and more interesting, it has been reported effective to accelerate wound healing in a follow‐up study.^145^ DermFactor® is a silicate‐based wound dressing widely used for wound management in burned skin, diabetic foot ulcers, and bedsores. A clinical study of DermFactor® has been conducted to evaluate its wound healing efficacy after anal surgery.^146^ It is found that the postoperative wound healing was significantly accelerated after the treatment of DermFactor®, which did not induce any adverse reaction to the patients. These clinical outcomes suggest that the bioceramic‐based biomaterials can be suitable wound dressing in practical clinical applications.

Although with many successes as we reviewed, current research on bioceramic‐based wound healing materials is far from mature and there are still many challenges ahead in developing bioceramic materials and their practical applications in skin tissue regeneration. Firstly, the physicochemical and biological properties of the final wound dressing products are dependent on the selection of bioceramic materials and the corresponding fabrication methods of the wound dressings. Current bioceramic‐based wound dressings are mostly prepared by simple physical blending methods. The covalent bonds between the bioceramic particles and the dressing matrix are rarely considered. In addition, the distribution and stability of bioceramics within the matrix are hardly under control. New fabrication technologies should be developed by taking advantage of the interactions between bioceramic ions and matrix molecules, which is important for tailoring whole material systems for practical wound healing applications. Besides, more multifunctional bioceramic‐based wound dressings should be designed with optimized multiple compositions, since it is difficult to impart multiple functionalities using a single‐component system in practical applications. Furthermore, more attention should also be paid to revealing the underlying mechanisms of both the single ion‐mediated bioactivity and the synergistic effect of different ion combinations on their regulation of different types of cells, which will ensure further progress on bioceramic‐based materials and offer new research opportunities in wound healing applications. Last but not least, the long‐term in vivo safety certification of bioceramic materials should be systematically demonstrated before consideration for practical applications in clinics. Overall, the above issues will be addressed with the tremendous efforts made in biomaterials for tissue engineering. The bioceramic materials with ion‐mediated multifunctionalities are believed to be exploited to overcome current challenges in wound healing applications and ultimately achieve clinical transformation.

## AUTHOR CONTRIBUTIONS

Xiaocheng Wang: Conceptualization; Investigation; Methodology; Funding acquisition; Project administration; Resources; Supervision; Writing – original draft; Writing – review & editing. Min Tang: Investigation; Methodology; Formal analysis; Writing – review & editing.

## CONFLICT OF INTEREST

The authors declare no conflict of interest.
